# Amixicile, a novel strategy for targeting oral anaerobic pathogens

**DOI:** 10.1038/s41598-017-09616-0

**Published:** 2017-09-05

**Authors:** Justin A. Hutcherson, Kathryn M. Sinclair, Benjamin R. Belvin, Qin Gui, Paul S. Hoffman, Janina P. Lewis

**Affiliations:** 1 Philips Institute of Oral Health Research, Virginia Commonwealth University, Richmond, Virginia USA; 2 Department of Microbiology and Immunology, Virginia Commonwealth University, Richmond, Virginia USA; 3 Department of Biochemistry, Virginia Commonwealth University, Richmond, Virginia USA; 4 Department of Medicine, Division of Infectious Diseases and International Health, University of Virginia, Charlottesville, Virginia USA

## Abstract

The oral microflora is composed of both health-promoting as well as disease-initiating bacteria. Many of the disease-initiating bacteria are anaerobic and include organisms such as *Porphyromonas gingivalis*, *Prevotella intermedia*, *Fusobacterium nucleatum*, and *Tannerella forsythia*. Here we investigated a novel therapeutic, amixicile, that targets pyruvate:ferredoxin oxidoreductase (PFOR), a major metabolic enzyme involved in energy generation through oxidative decarboxylation of pyruvate. PFOR is present in these anaerobic pathogenic bacteria and thus we hypothesized that amixicile would effectively inhibit their growth. In general, PFOR is present in all obligate anaerobic bacteria, while oral commensal aerobes, including aerotolerant ones, such as *Streptococcus gordonii*, use pyruvate dehydrogenase to decarboxylate pyruvate. Accordingly, we observed that growth of the PFOR-containing anaerobic periodontal pathogens, grown in both monospecies as well as multispecies broth cultures was inhibited in a dose-dependent manner while that of *S*. *gordonii* was unaffected. Furthermore, we also show that amixicile is effective against these pathogens grown as monospecies and multispecies biofilms. Finally, amixicile is the first selective therapeutic agent active against bacteria internalized by host cells. Together, the results show that amixicile is an effective inhibitor of oral anaerobic bacteria and as such, is a good candidate for treatment of periodontal diseases.

## Introduction

Periodontitis is an oral inflammatory disease that affects half of the United States adult population and disproportionally affects the aging population^1, 2^. It is initiated by bacteria that trigger the host inflammatory response, eventually leading to irreversible bone resorption and tooth loss. Although the oral microbiota consists of over 700 bacterial species, only a small portion leads to disease development and progression^3, 4^. In a healthy oral condition, the majority of bacterial species are Gram positive, such as *Streptococcus* sp. Once this population shifts towards a more dominant Gram negative anaerobic population, disease progression begins with gingivitis and continues towards more severe periodontal diseases^5, 6^. Among the periodontitis-associated bacteria are *Porphyromonas gingivalis*, *Tannerella forsythia*, and *Treponema denticola*, which are part of the so-called red complex^7–11^. Other bacteria contributing to periodontal disease include *Prevotella intermedia*, *Aggregatibacter actinomycetemcomitans*, and *Fusobacterium nucleatum* ^12, 13^. Bacteria present in the oral cavity form dental plaque that is mainly a complex microbial biofilm capable of withstanding a variety of mechanical and host defense mechanisms. In addition to colonizing the host in the form of oral biofilms, the bacteria can also be found in saliva, inhabiting the periodontal pocket which is rich in crevicular fluid, as well as attached and internalized by host cells. The latter shields the bacteria from immune defenses, as well as serves as a reservoir for recurrent infections.

Methods for treatment of periodontal disease rely mainly on antimicrobial therapy and include scaling and root planing, followed by antibiotic treatment. The use of antibiotics following surgical therapy has been shown to reduce periodontal pathogen burden when compared to mechanical methods alone or placebo^14^. Although effective, antibiotic therapy is still problematic. Firstly, most of the antibiotics currently used (such as clindamycin, tetracycline, and amoxicillin) are broad spectrum and thus eliminate both “health-promoting” and “disease-promoting” bacteria^15–19^. This leaves the treated oral environment ripe for re-infection by periodontal disease-promoting pathogens, as they do not have to compete with resident microflora. Secondly, the semi-selective antimicrobial metronidazole, although specifically inhibiting growth of anaerobic bacteria, is not very commonly used in clinic as it may cause serious side effects such as nausea, neuropathies, and colitis and there are issues with compliance^19–22^. Finally, there are concerns with repeated antibiotic interventions that over time will contribute to antimicrobial drug resistance and to dysbiosis, not only for the oral microflora, but also for the gut microbiome, a noted risk factor for development of *Clostridium difficile* colitis. Thus, there is an urgent need to identify more pathogen-selective drug targets and therapeutics that can overcome these limitations. Such therapeutics could also be used for preventive alteration of the microbiome that would keep at bay the members of pathogenic bacteria, thus preventing onset and progression of periodontal disease.

A promising, druggable target that would selectively affect the disease-promoting bacteria is pyruvate:ferredoxin oxidoreductase (PFOR). PFOR catalyzes the conversion of pyruvate and coenzyme A (CoA) to CO_2_ and acetyl-CoA and is an important component of many metabolic pathways found in anaerobic bacteria and parasites. Recently, the Hoffman laboratory developed a therapeutic, amixicile, which is a derivative of nitazoxanide (NTZ). NTZ is an FDA-approved therapeutic for the treatment of parasitic infections such as *Cryptosporidium*, *Trichomonas*, *Entamoeba*, and *Giardia* ^23, 24^ and shows *in vitro* activity against a wide range of strictly anaerobic bacteria^25, 26^. These small molecules inhibit the activity of PFOR by outcompeting pyruvate for binding to the thiamine pyrophosphate (TPP) cofactor of PFOR in the active site^27^. Studies in the Hoffman laboratory have shown that amixicile is a more potent and soluble derivative of NTZ that is superior in treating *Clostridium difficile* colitis when compared to the current mainline therapeutics^28, 29^.

Here we show that the oral anaerobic pathogens associated with periodontal disease are highly susceptible to amixicile and that commensal bacteria that do not express PFOR, such as *S*. *gordonii*, are unharmed. Importantly, amixicile was potent against these anaerobes under conditions simulating periodontal disease such as mixed broth cultures, biofilms, as well as in cell cultures. Finally, the action of amixicile was not ablated by the presence of 10% saliva or serum, lending support to potential efficacy in treatment of periodontal disease.

## Results

### The pyruvate:ferredoxin oxidoreductase (PFOR) pathway is conserved in many oral pathogens

**Figure 1:**
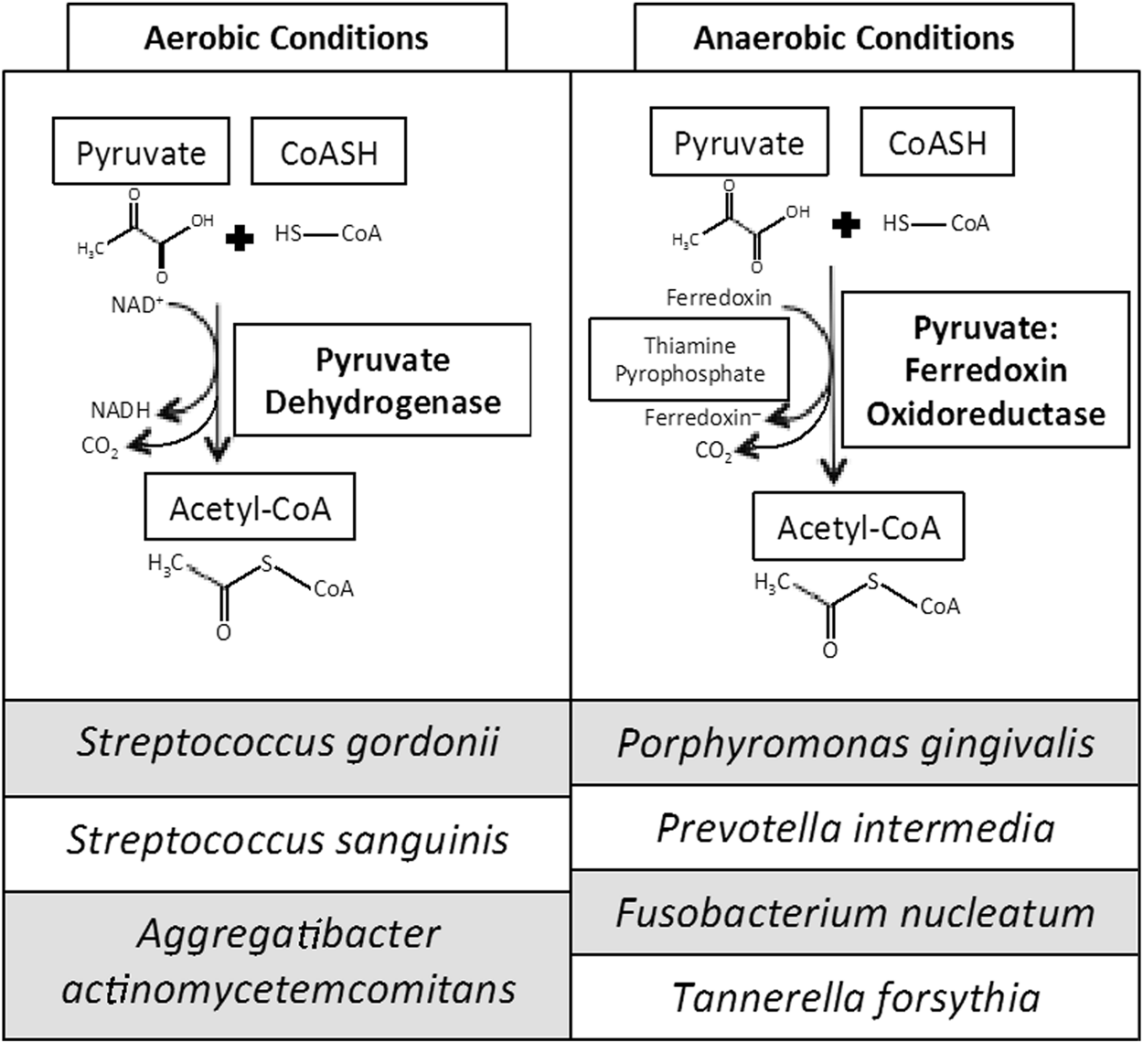
Pyruvate metabolism in aerobic and anaerobic bacteria. Under aerobic conditions, bacteria utilize pyruvate dehydrogenase to convert pyruvate into acetyl-CoA and carbon dioxide. Anaerobic bacteria can only grow under reduced oxygen concentrations and use pyruvate: ferredoxin oxidoreductase to convert pyruvate into acetyl-CoA and carbon dioxide.

The Kyoto Encyclopedia of Genes and Genomes (KEGG) database was used to determine the presence of genes coding for proteins involved in pyruvate catabolism within bacterial strains of interest. The genomes of oral pathogenic bacteria, *P. gingivalis*, *P. intermedia*, *F. nucleatum*, and *T. forsythia*, were found to contain genes encoding pyruvate:ferredoxin oxidoreductase (PFOR) (Fig. 1). Similarly, genomes of other anaerobic pathogens, *Clostridium perfringes* and microaerophile *Helicobacter pylori*, also contain this pathway. Oral facultative and aerotolerant anaerobic bacteria such as *A. actinomycetemcomitans* and *S. gordonii* express pyruvate dehydrogenase (PDH), which is also found in humans (Fig. 1). These data suggest that *P. gingivalis*, *P. intermedia*, *F. nucleatum*, and *T. forsythia* should be susceptible to amixicile, whereas* A. actinomycetemcomitans* and* S. gordonii* would not, as they do not contain the amixicile drug target, PFOR.

### Bioinformatic analysis of PFOR in oral bacteria

To further verify the possibility that amixicile may inhibit PFOR present in oral anaerobic bacteria, we performed bioinformatic analysis of the PFOR protein sequences present in the bacteria of interest. The crystal structure of PFOR from *Desulfovibrio africanus* has been published^30–32^. We used that data to gain insight into the structural features of the protein and to what extent the structure is conserved among members of the PFOR family.

**Figure 2:**
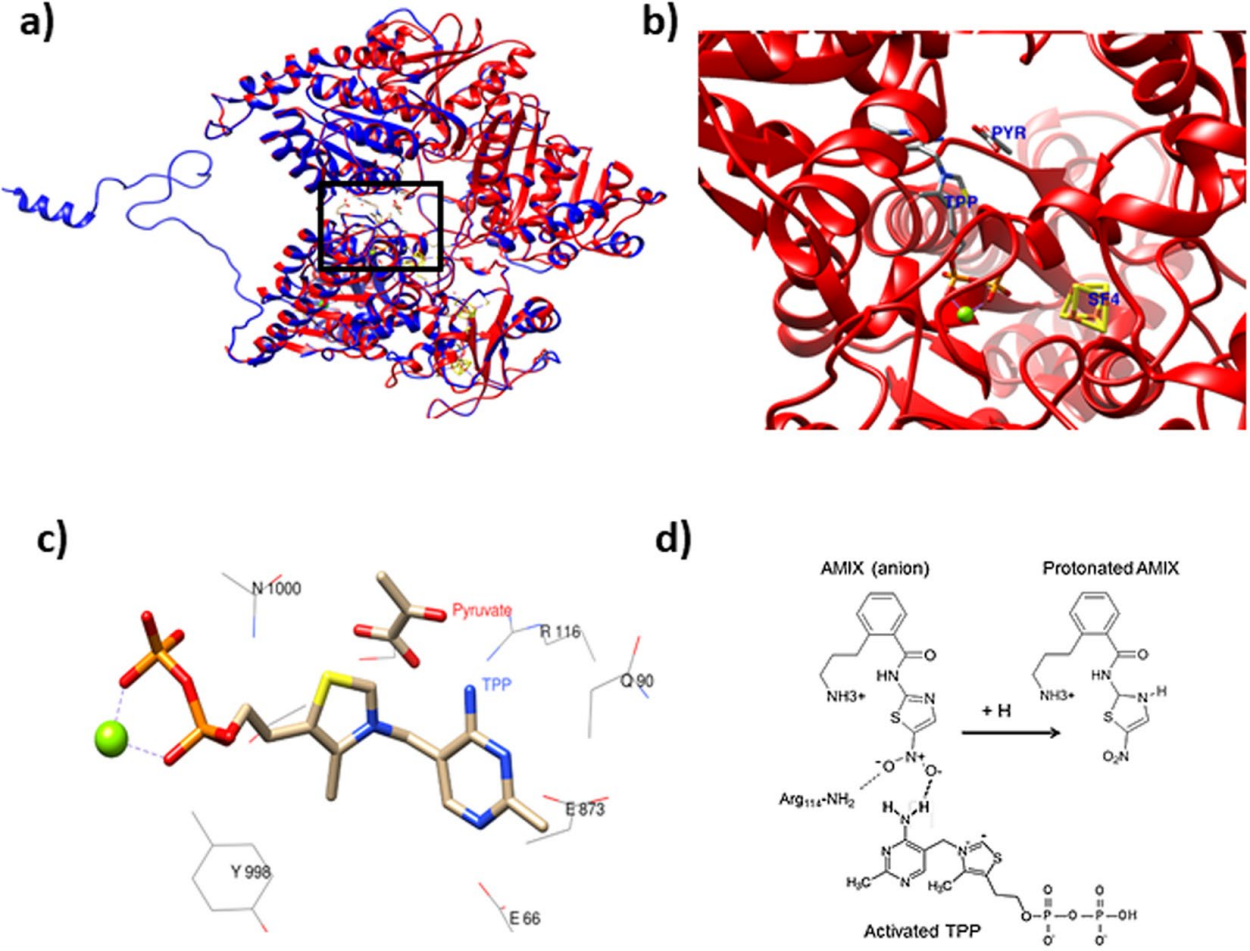
Conservation of the pyruvate:ferredoxin/flavodoxin oxidoreductase. (**a**) Overlay of the *Desulfovibrio africanus* crystal structure (PDB id: 2C3O) PFOR (blue) and homology model of *P*. *gingivalis* PFOR (red) monomer forms of the protein. The overlaid structures share an RMSD of 0.189 Å. The active site of the enzyme is highlighted by a black box. **(b)** The active site of the *P*. *gingivalis* PFOR where the enzyme catalyzes the interconversion of pyruvate and Acetyl-CoA. **(c)** Position and orientation of the TPP cofactor and pyruvate in the active site is maintained by conserved residues. (**d**) Proton abstraction mechanism of amixicile. The 5-nitro group of amixicile (AMIX) mimics the carbonyl carbon of pyruvate in forming a hydrogen bond with the aminopyrimidine of TPP. The highly conserved Arg114 of PFOR is predicted to also interact with the nitro group. In the process, anionic AMIX becomes protonated and NMR studies show that the proton localizes to the nitro or the ring nitrogen. TPP is now inactive until PFOR recycling reactivates the carbanion on the thiazole ring. Protonated AMIX loses the proton to water and the anion is restored.

In Table 1, we show that there is over 50% identity between the amino acid sequences across the family when compared pairwise. As an example, the PFOR structures between *D*. *africanus* and *P*. *gingivalis* are highly conserved (Fig. 2a,b). Importantly, the TPP binding domain is highly conserved among PFORs as catalytic function is dependent on TPP being maintained in a V shape^33^. The Glu66 is a conserved residue that is important for stabilization of the ylide conformation necessary for formation of the C2 carbanion on the thiazolium ring (Fig. 2c). In this configuration, the N4′ aminopyrimidine moiety is positioned close to the C2 carbanion of the thiazole, thus favoring interaction with the carbonyl carbon of pyruvate (Fig. 2c). Amixicile and parent compound NTZ attack this activated TPP complex. Modeling studies (Fig. 2d) reveal that the anionic nitro group of amixicile competes with pyruvate for the protonated N4′ amino group of TPP^27^. Kinetic studies had previously shown that the K_m_ for amixicile and NTZ is over two orders of magnitude lower than the K_m_ for pyruvate^23, 24^. Deprotonation of the N4′ aminopyrimidine by amixicile inactivates the catalytic cycle of PFOR. Protonation temporarily inactivates amixicile, but the proton is rapidly lost to water (pKa 6.2) in reactivation of the anionic state of amixicile^27^. Given the catalytic mechanism and high degree of conservation among PFORs, we predicted that amixicile would show selective toxicity for the oral anaerobes^27^.

**Table 1:** Identity (%) of PFOR present in anaerobic bacterial species.

	*D. africanus*	*T. denticola*	*C. difficile*	*F. nucleatum*	*P. gingivalis*	*T. forsythia*
*T. denticola*	56.7					
*C. difficile*	59.1	64.3				
*F. nucleatum*	56.3	59.5	59.7			
*P. gingivalis*	56.0	57.6	60.7	62.1		
*T. forsythia*	55.3	56.3	60.0	62.1	72.6	
*P. intermedia*	52.1	55.1	57.0	56.8	67.7	74.4

### Amixicile inhibits growth of anaerobic bacteria

**Figure 3:**
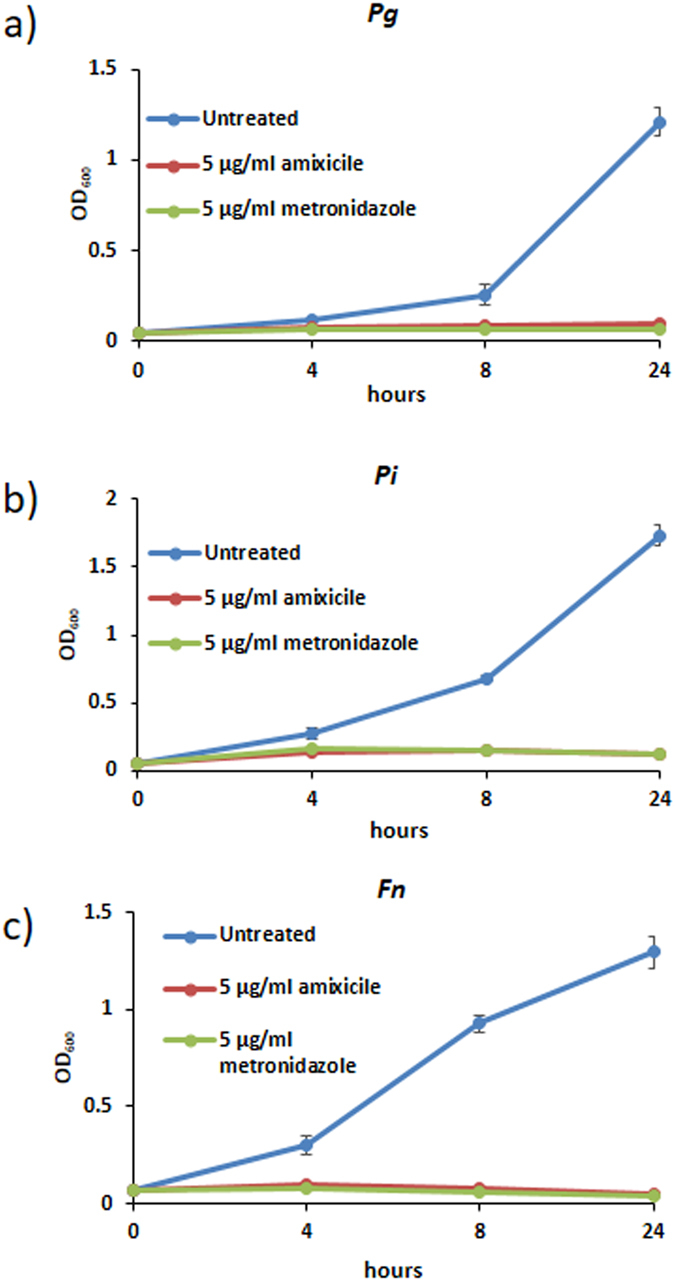
Effect of amixicile on growth of anaerobic bacteria. Overnight cultulres of a) *P. gingivalis* W83, b) *P. intermedia* 17 and c) *F. nucleatum* 25586 were inoculated into BHI broth containing either amixicile (5 µg/mL) or metronidazole (5 µg/mL) to an OD_600_ of 0.05 under anaerobic conditions. Cultures grown in BHI without antibiotics (untreated) served as control. The bacteria were grown for 24 hr and growth of the bacteria was monitored by measuring OD_600_ at 0, 4, 8 and 24 hr. Means and SD from two biological replicates performed in technical triplicates are shown.

**Figure 4:**
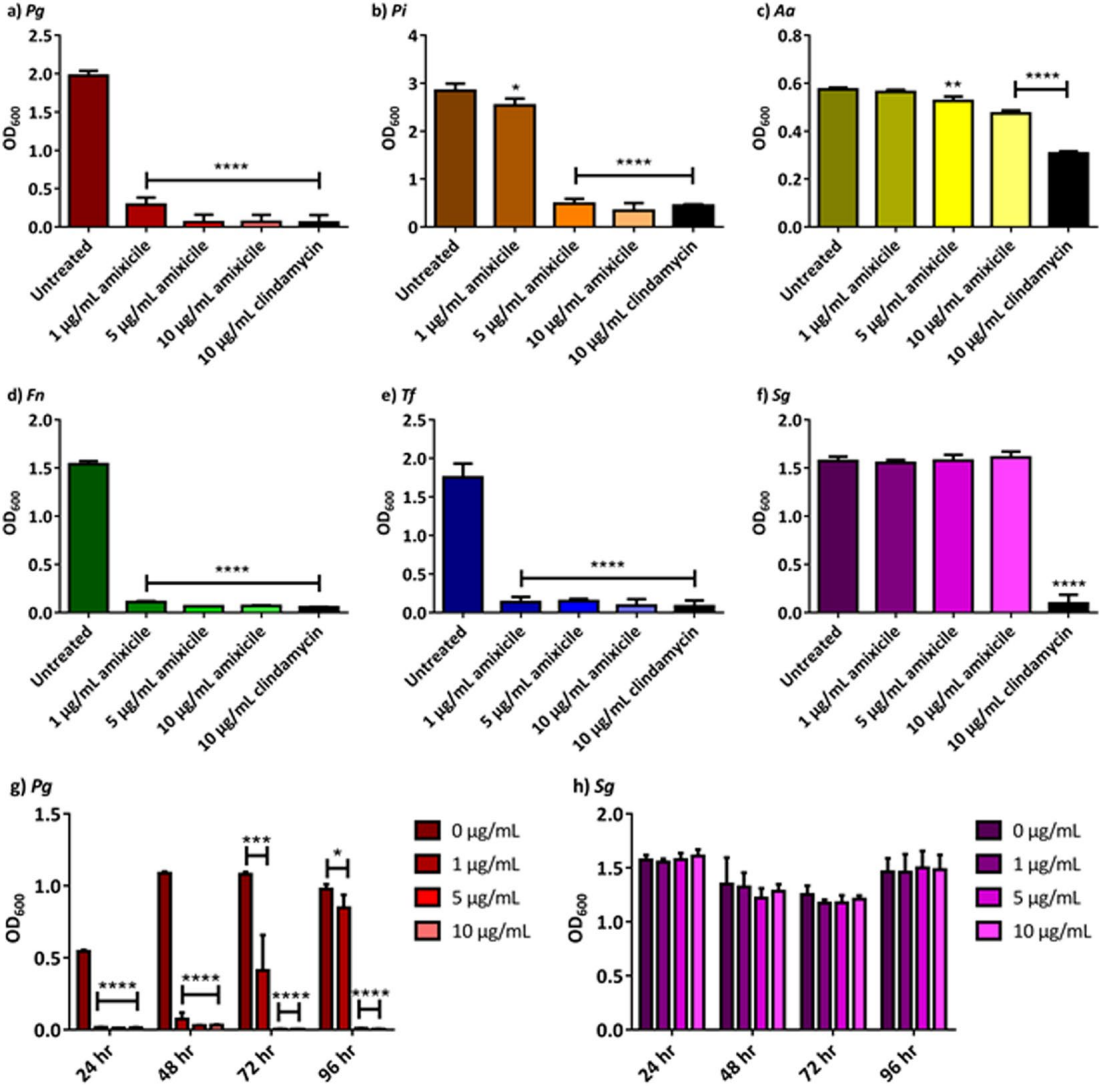
Amixicile inhibits growth of anaerobic bacteria. Bacterial strains: (**a**) *P*. *gingivalis* W83, **(b)** *P*. *intermedia* 17, **(c)** *A*. *actinomycetemcomitans* 33384, (**d**) *F*. *nucleatum* 25586, (**e**) *T*. *forsythia* 43037, and (**f**) *S*. *gordonii* 10558 were cultured in BHI broth with or without amixicile (1, 5, and 10 µg/mL) and the OD_600_ was measured at 24 hr. Clindamycin (10 µg/mL) was used as a positive control. (**g**) *P*. *gingivalis* and (**h**) *S*. *gordonii* were cultured in BHI broth containing various concentrations of amixicile (0, 1, 5 and 10 µg/mL) and the microbial growth was monitored over 96 hr. Results are expressed as mean ± SD. Statistical analysis was done by ANOVA. *p < 0.0332, **p < 0.0021, ***p < 0.0002 and ****p < 0.0001 compared to the untreated control.

We first examined the effect of amixicile on the growth of oral anaerobic pathogens associated with periodontal disease. As shown in Fig. 3, amixicile inhibited growth of *P*. *gingivalis* W83, *P*. *intermedia* 17, and *F*. *nucleatum* 25586. The inhibitory effect was similar to that of metronidazole, a known antibiotic active against anaerobic bacteria. From those studies we continued using the antibiotic concentration of 1 µg/mL, or even higher for biofilms and host-pathogen interactions (5 µg/mL), to ensure that growth of *P*. *intermedia* was inhibited. Thus, bacterial strains were grown anaerobically for 24 hr with 0, 1, 5 or 10 µg/mL of amixicile or clindamycin. *T*. *forsythia* was grown for 96 hr due to its slow growth rate. As shown in Fig. 4, growth of *P*. *gingivalis*, *P*. *intermedia*, *F*. *nucleatum*, and *T*. *forsythia* was inhibited in the presence of 1 µg/mL amixicile. A similar inhibition profile was seen with 5 µg/mL and 10 µg/mL amixicile. Clindamycin, used clinically to treat periodontal infection, also inhibited growth of the bacteria as expected. We also tested the susceptibility of *S*. *gordonii* to amixicile, since it lacks the drug target and utilizes PDH to metabolize pyruvate. As shown in Fig. 4e, growth of this bacterium was not affected by amixicile, indicating that this therapeutic specifically targets bacteria that use PFOR for energy generation. Another oral pathogen, *A*. *actinomycetemcomitans*, which also uses PDH for pyruvate metabolism (Fig. 1) was slightly inhibited at 5 and 10 µg/mL amixicile (Fig. 4c). Additional experiments were conducted to verify growth inhibition in cultures incubated for a prolonged period of time. *P*. *gingivalis* and *S*. *gordonii* were grown for 96 hr with 0, 1, 5 or 10 µg/mL amixicile. Growth yields, as shown in Fig. 4g,h, were similar at all times monitored: 24, 48, 72 and 96 hr. Finally, dose-dependent inhibition was done using concentrations of amixicile ranging from 0-10 µg/mL to determine the minimal inhibitory concentration (MIC) for each bacterium. *F*. *nucleatum* and *T*. *forsythia* were susceptible to 0.5 µg/mL, *P*. *gingivalis* was affected by 1 µg/mL, and *P*. *intermedia* was inhibited at 2 µg/mL (Supplemental Fig. 1). Growth of *A*. *actinomycetemcomitans* and *S*. *gordonii* was not affected by concentrations of amixicile as high as 3 µg/mL, indicating that the bacteria are not sensitive to that therapeutic.

In conclusion, amixicile at 1 µg/mL significantly inhibited growth of a variety of oral anaerobic bacteria up to 96 hr (as shown using *P*. *gingivalis*) but had no effect on PFOR-lacking bacteria, such as *S*. *gordonii*, at any of the time points measured (Fig. 4g,h).

### Amixicile specifically inhibits growth of anaerobic bacteria in multispecies cultures

**Figure 5:**
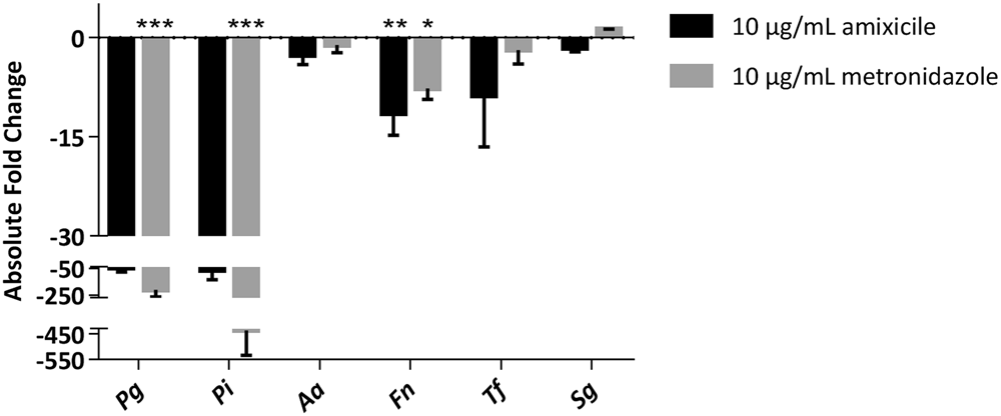
Amixicile inhibits oral pathogens in multispecies broth culture. Bacterial strains were inoculated in BHI broth at an OD_600_ of 0.05. Broth cultures contained 10 µg/mL amixicile, 10 µg/mL metronidazole, or were left unsupplemented. 1 OD_600_ of each culture was harvested at 48 hr and quantified by qPCR. *Pg* – *P*. *gingivalis* W83, *Pi* – *P*. *intermedia* 17, *Aa* – *A*. *actinomycetemcomitans* 33384, *Fn* – *F*. *nucleatum* 25586, *Tf* – *T*. *forsythia* 4303l7, *Sg* – *S*. *gordonii* 10558. Results are expressed as mean ± SD compared to the untreated control from experiments performed in triplicate. Statistical analysis was done by ANOVA. *p < 0.0332, **p < 0.0021, ***p < 0.0002 and ****p < 0.0001 compared to the untreated control.

To investigate a more clinically relevant model, multispecies broth cultures were used to examine the effect of amixicile on growth inhibition of the PFOR-containing bacteria. Equivalent amounts of *P*. *gingivalis*, *P*. *intermedia*, *A*. *actinomycetemcomitans*, *F*. *nucleatum*, *T*. *forsythia*, and *S*. *gordonii* were used to inoculate a multispecies broth culture and growth was monitored for 48 hr. The yield of mixed species cultures was monitored in the presence of amixicile and compared to those incubated with metronidazole or grown without antibiotics. To determine specific inhibition of the anaerobic bacteria in the multispecies cultures, DNA was isolated from the cultures and levels of bacteria were determined using qPCR analysis. The analysis has shown that amixicile inhibited growth when compared to the untreated control group of all PFOR-containing bacteria: *P*. *gingivalis*, *P*. *intermedia*, *F*. *nucleatum*, and *T*. *forsythia* (Fig. 5). Similar results were obtained using metronidazole. In contrast, neither antimicrobial affected growth of *S*. *gordonii* (Fig. 5), indicating selectivity for both therapeutics. Also, the growth of *A*. *actinomycetemcomitans* was inhibited only in the presence of high concentrations of amixicile (5 µg/mL and 10 µg/mL) (Figs 4 and 5). These data demonstrate that amixicile is an effective inhibitor of growth of *P*. *gingivalis*, *P*. *intermedia*, *F*. *nucleatum*, and *T*. *forsythia* when the bacteria are grown in multispecies cultures.

### Saliva and serum do not reduce the inhibitory activity of amixicile

**Figure 6:**
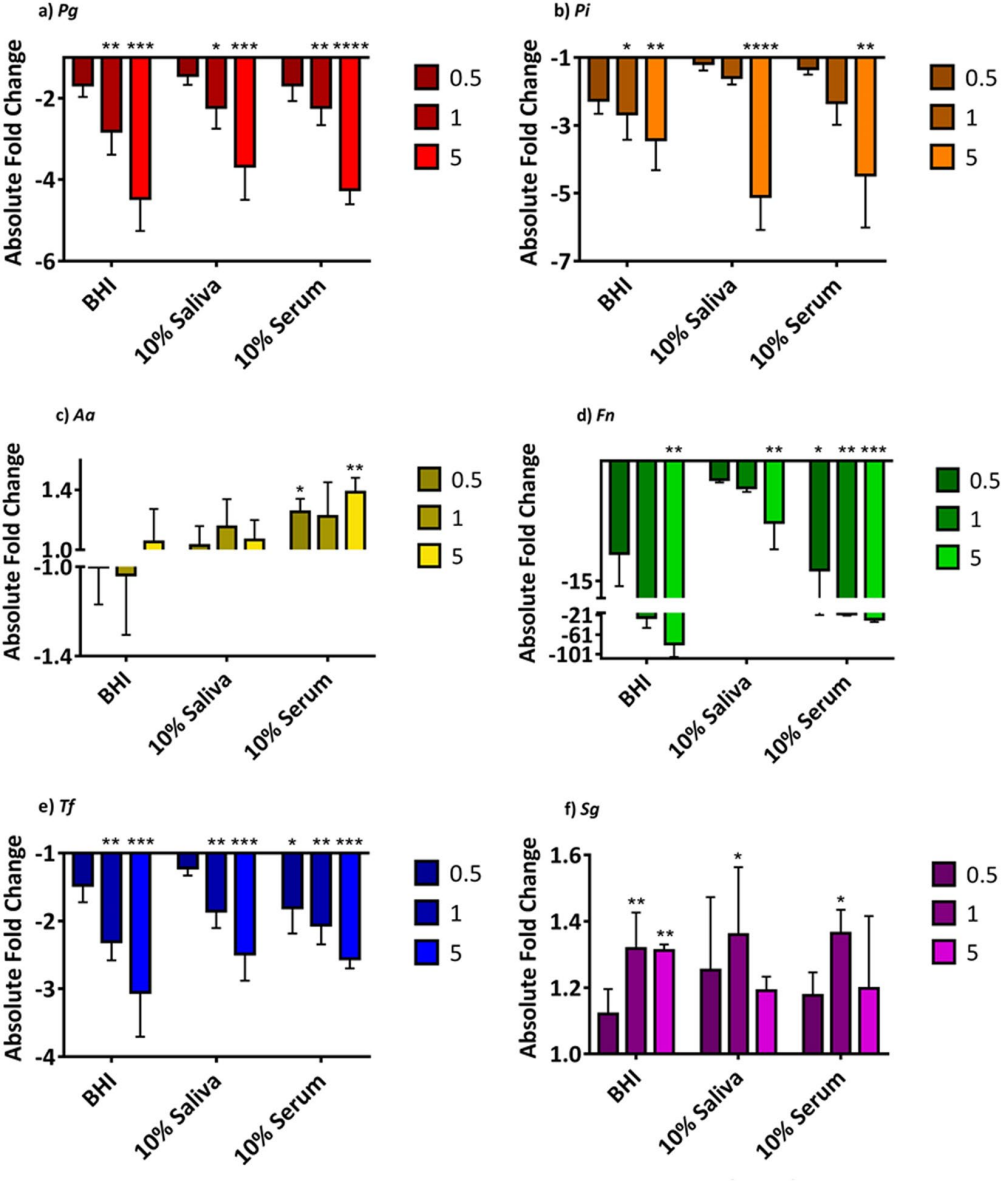
Effect of saliva and serum on the inhibitory activity of amixicile. Bacterial strains: (**a**) *P*. *gingivalis* W83, **(b)** *P*. *intermedia* 17, **(c)** *A*. *actinomycetemcomitans* 33384, (**d**) *F*. *nucleatum* 25586, (**e**) *T*. *forsythia* 43037, and (**f**) *S*. *gordonii* 10558. The absolute fold change between amixicile-treated bacteria and controls grown in different media were determined by qPCR. Multispecies cultures were grown anaerobically overnight in BHI or BHI supplemented with either 10% filtered human saliva or 10% filtered human serum in the presence of 0 (control), 0.5, 1 or 5 μg/mL amixicile. Genomic DNA was isolated from cultures grown for 24 hr and qPCR was run with 16s species-specific primers. Results are expressed as mean ± SD of amixicile-treated sample compared to the untreated control from experiment performed in triplicate. Statistical analysis was done by ANOVA. *p < 0.0332, **p < 0.0021, ***p < 0.0002 and ****p < 0.0001 compared to the untreated control.

To determine the effect of more *in vivo*-like conditions, we tested the inhibitory activity of amixicile on bacteria grown in the presence of 10% saliva or serum. The multispecies cultures were prepared as above and levels of bacteria were determined using qPCR. The qPCR results shown in Fig. 6 with the *A*. *actinomycetemcomitans* and *S*. *gordonii* primers showed no significant reduction in growth resulting from the presence of amixicile in any of the media tested when compared to the untreated control. Growth of *A*. *actinomycetecomitans* was even upregulated in saliva and serum. While statistically significant, less than 2-fold upregulation of *S*. *gordonii* was observed compared to the untreated control. This is expected, given that *A*. *actinomycetemcomitans* and *S*. *gordonii* are not susceptible to amixicile. *P*. *gingivalis*, *P*. *intermedia*, and *T*. *forsythia* had similar growth patterns in BHI, 10% saliva, and 10% serum. *F*. *nucleatum* showed a higher susceptibility to amixicile in BHI than in 10% saliva or 10% serum but it was still significantly affected in all three media tested. These results indicate that amixicile should be effective in reducing bacterial growth in the oral cavity where either saliva or serum are present.

### Amixicile prevents maturation of *P*. *gingivalis* biofilms, but not of *S*. *gordonii*

**Figure 7:**
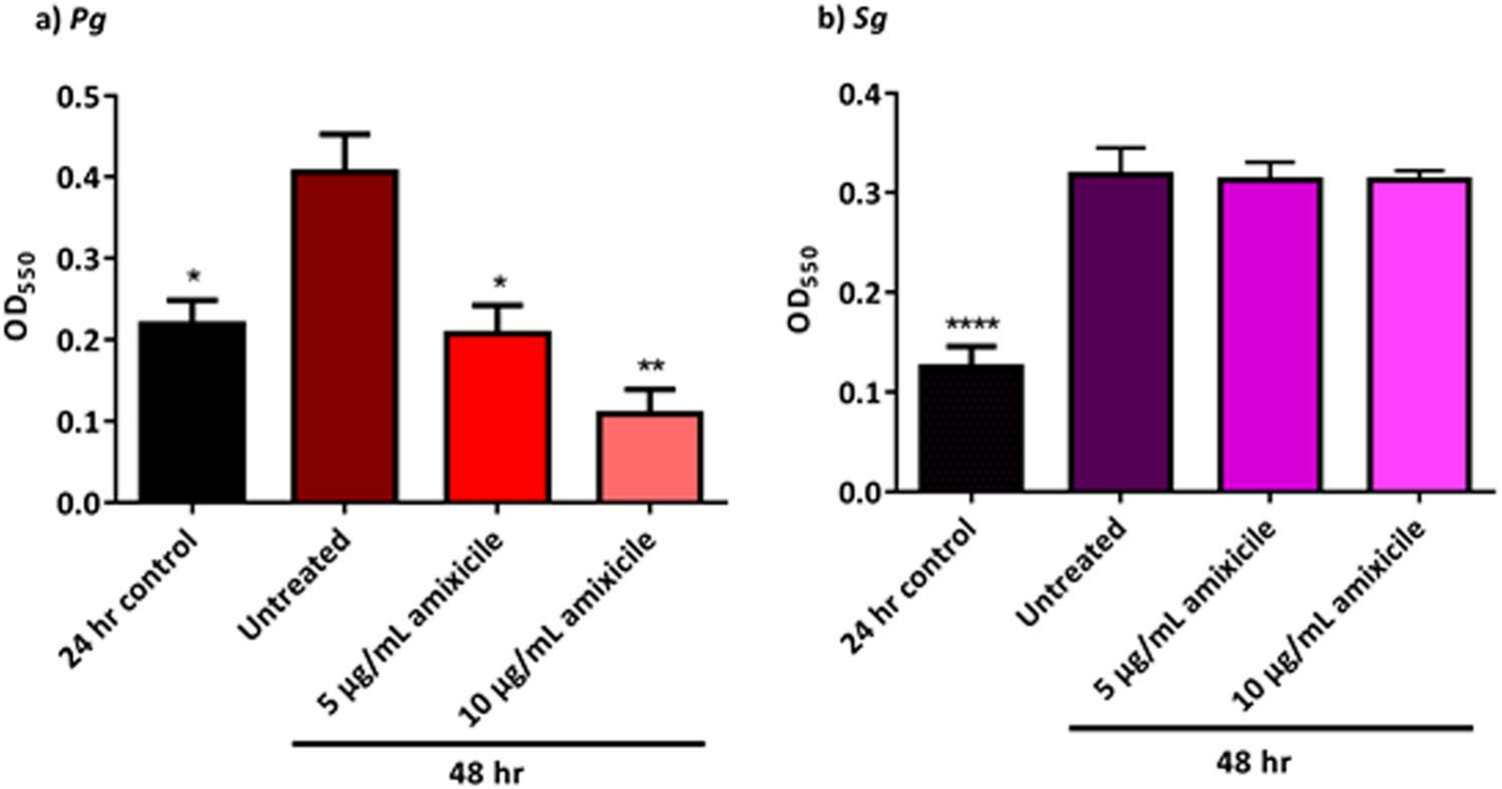
*P. gingivalis* monospecies biofilm maturation is inhibited by amixicile. (**a**) *P*. *gingivalis* 33277 and **(b)** *S*. *gordonii* 10558 biofilms were cultured in BHI for 24 hr. After 24 hr, fresh media containing 0, 5, or 10 µg/mL amixicile was added and the biofilms were incubated for an additional 24 hr. Biofilm volumes were measured by crystal violet staining at OD_550_. Results were compared to the untreated 48 hr control group. All experiments were performed in triplicate. Statistical analysis was done by ANOVA. *p < 0.0332, **p < 0.0021, ***p < 0.0002 and ****p < 0.0001 compared to the untreated control.

Oral bacteria form biofilms known as dental plaque that aid in bacterial survival within the host. Therefore, we cultured *P*. *gingivalis* and *S*. *gordonii* in the form of monospecies biofilms for 24 hr and treated the biofilms with amixicile for an additional 24 hr. Our results show that the level of biofilm formation for the 5 µg/mL amixicile-treated *P*. *gingivalis* after 48 hr is comparable to that of the 24 hr control showing the level of biofilm prior to amixicile addition (Fig. 7). Furthermore, a higher dose of amixicile (10 µg/mL) reduced the biofilm volume after 24 hr treatment when compared to the control biofilm (Fig. 7). As expected, amixicile did not have an effect on the volume of *S*. *gordonii* biofilms as the levels of the biofilm nearly doubled despite the presence of amixicile when compared to the 24 hr control biofilm. These results suggest that amixicile can be used to inhibit growth and prevent formation of biofilms by *P*. *gingivalis* while not affecting the growth and biofilm formation of primary colonizers such as *S*. *gordonii*.

### Amixicile selectively inhibits anaerobic bacteria in multispecies biofilms

**Figure 8:**
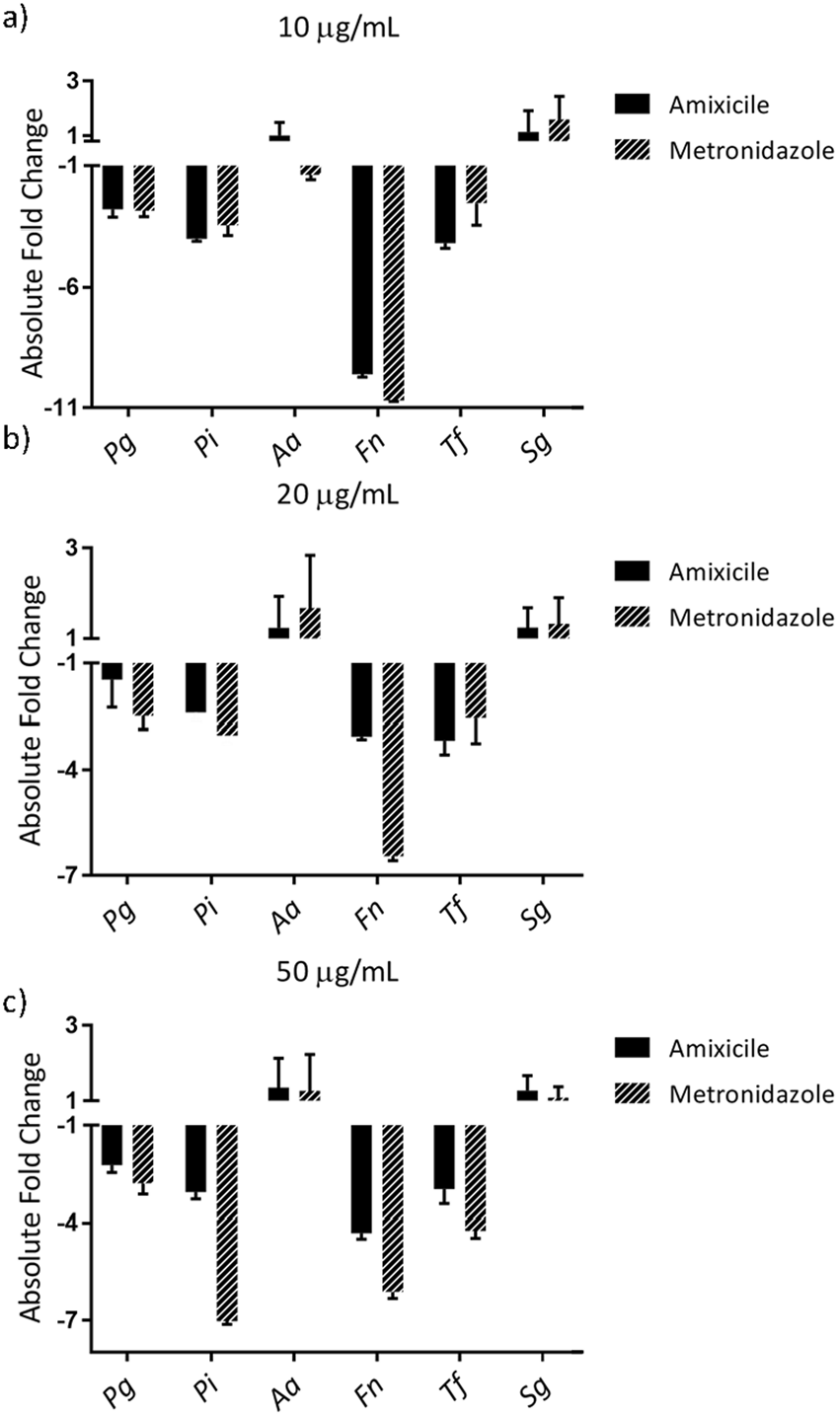
Amixicile selectively inhibits growth of anaerobes in a multispecies biofilm model. Four day-old multispecies biofilms were treated with 10 µg/mL a), 20 µg/mL b), and 50 µg/mL c) amixicile or metronidazole. Untreated biofilms served as controls. The biofilms were incubated for five additional days with daily growth media replacement. Bacteria were then collected and DNA was harvested for qPCR analysis. Results shown are mean ± SD compared to the untreated control from an experiment performed in triplicate. *Pg* – *P*. *gingivalis* 33277, *Pi* – *P*. *intermedia* 17, *Aa* – *A*. *actinomycetemcomitans* 33384, *Fn* – *F*. *nucleatum* 25586, *Tf* – *T*. *forsythia* 43037, *Sg* – *S*. *gordonii* 10558.

Oral biofilms are comprised of many different species of bacteria; therefore we tested the effectiveness of amixicile against a multispecies *in vitro* biofilm model. Four day-old biofilms were treated with amixicile, metronidazole, or left untreated for five additional days. Biofilm DNA was harvested for qPCR analysis by 16S gDNA. Results show that amixicile and metronidazole inhibit *P*. *gingivalis*, *P*. *intermedia*, *F*. *nucleatum*, and *T*. *forsythia* levels in the biofilm starting with 10 µg/mL of amixicile, but *A*. *actinomycetemcomitans* and *S*. *gordonii* were unaffected (Fig. 8). This suggests that amixicile may potentially be a valuable tool in combating oral biofilms *in vivo* by selectively inhibiting many oral pathogens.

### Live/dead staining of bacteria exposed to amixicile

**Figure 9:**
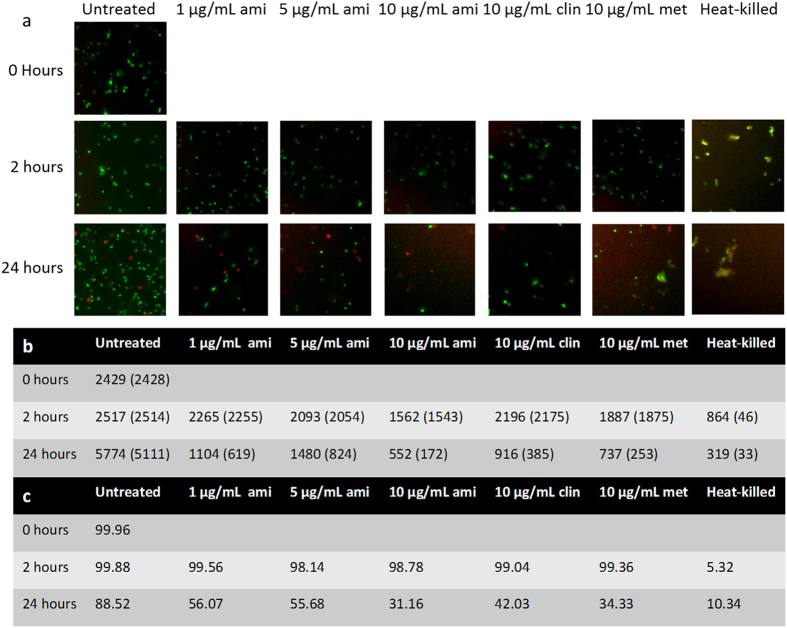
Live/dead staining to determine the mechanism of amixicile. (**a**) *P. intermedia* was grown for up to 24 hr with amixicile, clindamycin, metronidazole, or without antimicrobial. Representative fluorescence microscopy images are shown. The green stain indicates live cells. Red or yellow color indicates dead cells. Cells were manually counted as live or dead. **(b)** Total number of cells, with the number of live cells in parentheses counted for this experiment. **(c)** Percentage of live cells.

Amixicile inhibits the activity of PFOR thus reducing energy generation by the bacteria. To determine the bactericidal or bacteriostatic action of amixicile, we performed staining for live/dead bacteria. As shown in Fig. 9, as the dosage of amixicile increased, the total number of bacterial cells decreased. The green stain represents live cells while the red stain indicates dead cells, since propidium iodide cannot break through an intact cell membrane. Occasionally the stains overlap, particularly as seen in the heat-killed samples serving as a control for our studies, showing a yellow color, which also indicates dead cells. As the concentration of amixicile increased, both the total number of cells and the percentage of live cells decreased. Clindamycin was used as a bacteriostatic control and metronidazole and heat as bactericidal controls. All treatments, excluding heat, were bacteriostatic at early time points. The number of cells was reduced at 2 hr (Fig. 9a) but the cells were alive according to the green stain. At 24 hr, however, it was clear that amixicile is bactericidal as we detected cells that were stained in red. In conclusion, amixicile at high doses shows similar effectiveness to metronidazole.

### Colonization of eukaryotic cells by oral bacteria is inhibited by amixicile

**Figure 10:**
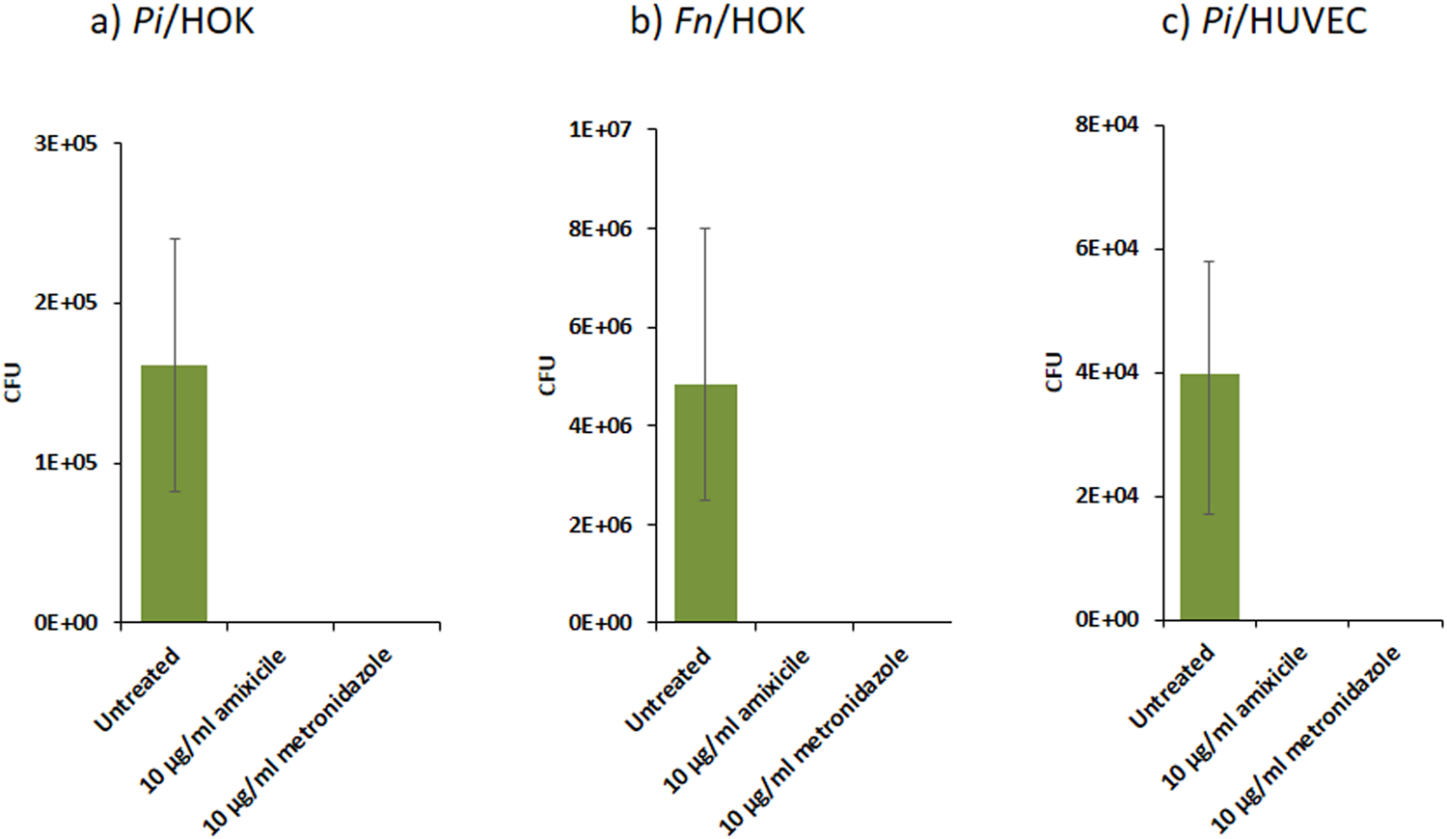
Amixicile inhibits bacterial colonization of HOKs and HUVECs. HOKs (**a** and **b**) and HUVECs (**c**) were challenged with oral bacteria (**a**,**c** - *P*. *intermedia* 17, **b** – *F*. *nucleatum*) at an MOI of 1:100 for 24 hr with and without the addition of amixicile or metronidazole. CFU counts of colonization at 24 hr as observed on blood agar plates seven days post-infection. Data from three biological replicates performed in duplicate are presented.

Many oral pathogens have adapted to the oral cavity by invading host cells to avoid destruction by the immune response^34^. Similarly, oral bacteria were found to invade a variety of other eukaryotic cells including the human umbilical cord endothelial cells (HUVECs)^27^. To determine if amixicile is effective against bacteria associated with host cells (both internalized and bound to host cells), monolayers of human oral keratinocytes (HOKs) and HUVECs were challenged with oral bacteria and then treated with amixicile, metronidazole, or left untreated. The infected eukaryotic cells were lysed at 24 hr post-treatment and viable bacteria (enumerated as CFUs) were determined according to a published protocol^35^. Cells treated with amixicile and metronidazole displayed significant reduction in the number of viable bacteria when compared to the untreated control group (Fig. 10). While in the presence of metronidazole, some live bacteria were still observed. No colonies were obtained using HOKs and HUVECs infected with *P*. *intermedia* or *F*. *nucleatum* when incubated in the presence of 10 µg/mL amixicile. This provides evidence that amixicile can affect both intra- and extracellular bacteria. These results were visually confirmed by confocal microscopic analysis (Supplemental Fig. 2). While bacteria were noted in the untreated HUVECs, no bacteria were detected in either the metronidazole- or amixicile-treated cells.

## Discussion

The oral microbiome is very dynamic and it can change its composition, eventually leading to either healthy or diseased states of the host, mainly through altering the abundance of bacterial species. Previous work has shown that a “healthy” microbiome is composed primarily of Gram positive bacteria such as streptococci, with a small proportion of pathogenic Gram negative anaerobes^6^. The shift towards the diseased microbiome involves acquisition of a larger proportion of Gram negative anaerobic bacteria such as *P*. *gingivalis* and *T*. *forsythia* that ultimately leads to activation of host response and development of periodontal disease (PD)^4, 36, 37^. Our bioinformatics analysis shows that PFOR is present in the pathogenic bacteria, while it is absent in the commensal streptococci. We performed our bioinformatics analysis on a limited number of bacteria, only those used in our study, and compared our results to bacteria in which PFOR was successfully targeted by amixicile, such as *Clostridium difficile* and *Helicobacter pylori* ^28, 38^. Our data sets the platform for a future, more comprehensive, systems biology approach, where all available genomes for oral bacteria will be scrutinized for the presence of PFOR.

We noted high conservation of the PFOR enzyme across genera, suggesting that this is an essential enzyme for the bacteria and required for proper metabolic activity. Specifically, many of the residues vital to the catalytic activity and coordination of both the pyruvate and TPP cofactor are conserved across all of the species we observed. This is fortunate as recent docking studies reveal that amixicile binds at the same site as pyruvate and interacts with the aminopyrimidine of the TPP^25^. Such similarity in the active site bodes well for the effectiveness of PFOR inhibition by amixicile in bacteria relying on the enzyme for energy production.

Our MIC studies show amixicile to be effective at inhibiting growth of *P*. *gingivalis*, *F*. *nucleatum*, and *T*. *forsythia* at 1 µg/mL. A higher dose, 5 µg/mL, was required to inhibit growth of *P*. *intermedia*. However, it is noteworthy that even at 10 µg/mL, no inhibition of *S*. *gordonii* is observed. This bacterium does not rely on the PFOR enzyme for energy generation and thus was expected to have a consistent growth rate unaffected by amixicile. *A*. *actinomycetemcomitans* also does not encode PFOR and was not expected to be inhibited by amixicile. However, approximately a 10% reduction in growth rate was observed with 10 µg/mL amixicile indicating that amixicile could possibly affect the growth of the bacterium through a non-PFOR-related mechanism. A slight effect of NTZ and amixicile on bacteria not expressing PFOR was reported previously^39, 40^.

Our data demonstrating that amixicile is effective in multispecies broth cultures indicates that the therapeutic will be effective in *in vivo* conditions where multiple bacteria are found. We used a dose of 10 µg/mL, which is equivalent to a dose of 500 mg for an approximately 130 lb patient. This dose was also effective to inhibit growth of the bacteria when grown as biofilms and thus would be relevant as such a dose is used clinically^40^. It is noteworthy that inhibition of PFOR-containing bacteria in the multispecies mixtures was also seen with 5 µg/mL (results not shown), further reinforcing that the bactericidal dosage will be applicable to an *in vivo* setting.

Our assumption is further reinforced by our studies performed in the presence of either saliva or serum, the two major factors that may affect the inhibitory activity of amixicile. *F*. *nucleatum* seemed to be the most susceptible to amixicile in our multispecies study in all three types of media tested. *T*. *forsythia*, although a slow-growing organism, still had significantly reduced growth rates in all media tested. *P*. *gingivalis* and *P*. *intermedia* were reduced less than expected based on the monospecies broth studies. Nevertheless, the 5 µg/mL of amixicile was an effective inhibitor of growth of those organisms in either saliva or serum. It has been shown previously that *S*. *gordonii* and other bacteria commonly found in the oral cavity can provide some protection for *P*. *gingivalis* and *P*. *intermedia* during a periodontal disease state and in times of stress. That may have evidenced itself in our multispecies cultures compared to the monospecies cultures as higher antibiotic dosage was required to inhibit growth of the anaerobes when the bacteria were grown in the six-species biofilm^41^.

Amixicile inhibits the growth of these pathogens at 1 µg/mL and completely abolishes growth at 5 and 10 µg/mL. Monospecies biofilm maturation is inhibited at 5 and 10 µg/mL amixicile. In these experiments, amixicile was tested against pre-formed biofilms. It has been shown that biofilms are more resistant to antibiotic action due to reduced penetration of antibiotics into the biofilm structures as well as an increase in the number of persister cells resistant to the antibiotic^42^. Therefore, a higher dose of antibiotic is required, in order for it to be effective against the layers of bacteria present in the biofilm. In multispecies biofilms particularly, many bacteria are protective against each other. It is noteworthy that *S*. *gordonii* growth, both in broth and biofilm maturation is not affected by amixicile at 5 or 10 µg/mL. This is a primary colonizer and thus a health-promoting bacterium.

Amixicile aids in reducing the colonization of oral bacteria in both HOKs and in HUVECs. Amixicile has been shown to be internalized by Caco-2 cell monolayers^38^ and this may explain why we observed the reduction of the live internalized bacteria. Most antibiotics are not taken up by eukaryotic cells and thus are not effective in reducing the intracellular microbial burden. Amixicile is thus unique in this aspect, as it will also reduce the levels of intracellular bacteria that alter host cell functions, as well as serve as a reservoir for future re-infections. This is highly significant considering the occurrence of recurrent infections due to the internalized bacteria that are not eliminated during standard periodontal therapy^17, 43^.

Another problem emerging in the treatment of oral infections is antibiotic resistance^44^. Antibiotic resistance develops as bacteria either mutate to avoid a drug’s mechanism of action or acquire the ability to export the antibiotic. Amixicile is highly novel in this regard, as the mechanism of action disrupted is the cofactor of PFOR, thiamine pyrophosphate, rather than the microbial enzyme itself, thus reducing the possibility of developing resistance to amixicile by mutation^25^. As noted in Kennedy *et al*.^25^, there have been no reports of drug resistance in the clinical use of nitazoxanide (NTZ), which might be an indicator of the potential longevity of this class of antimicrobials and the unique nature of mechanistic action.

Our studies show that amixicile is highly specific and affects bacteria that have the PFOR pathway. This is highly significant, as many bacteria that cause periodontal disease are indeed anaerobic and do encode PFOR. The therapeutic potential of amixicile may also have further use as the oral anaerobic bacteria also play a role in systemic diseases^45^ and even cancer^46, 47^. This is reinforced by recent findings that *H*. *pylori* is associated with higher numbers of periodontal pathogens, such as *P*. *gingivalis*, in the oral cavity, as well as is a major etiological agent in cancer development^48^. Targeting the pathogenic bacteria and sparing the beneficial ones will have significant potential not only for treatment of diseases but, most importantly, for preventive measures where the levels of pathogenic bacteria in microbiomes could be reduced, possibly converting such microbiomes to health-promoting ones.

Treatment of periodontal disease is primarily based on mechanical methods such as scaling and root planing to reduce of the microbial burden. Antibiotics are usually used as adjunct therapy in more severe cases due to their effect on the total microbial flora of the patient as well as the risk for development of antibiotic resistance. The antibiotics used are mostly broad-spectrum ones, thus affecting the total microbiome, both the aerobic health-promoting bacteria as well as the pathogenic anaerobic bacteria. However, several studies have shown that the use of metronidazole, a redox-active prodrug that is activated by PFOR and thus is effective against anaerobic bacteria, is as effective as mechanical methods (scaling and root planing) in treatment of periodontal disease^49–51^. Furthermore, metronidazole also reduced the need for periodontal surgery^52^. Such promising results of the use of antibiotic therapy in treatment of periodontal disease were halted by the toxic nature of metronidazole, as well as concerns about the development of antibiotic resistance^52^. Thus, metronidazole would not be a viable antibiotic for treatment of a widespread condition such as periodontal disease. Several attributes of amixicile, a novel antimicrobial targeting the anaerobic pyruvate:ferredoxin oxidoreductase (PFOR) and related enzymes, suggest it might be amenable to treatment of PD and include: (1) it is readily absorbed (systemic bioavailability), (2) it does not accumulate in or alter the gut microbiome, (3) it is well-tolerated (nontoxic) in animal models and (4) it has the ability to concentrate in areas of inflammation (serum leakage)^38^. The latter might prove beneficial to PD where concentration in crevicular fluid would be highly desired. Here we show that amixicile exhibits excellent *in vitro* activity against the oral anaerobes associated with PD. Although it has similar inhibitory activity to that of metronidazole in our *in vitro* studies, amixicile was more effective in eliminating bacteria in animal models of colitis^38^. Thus, based on the efficacy studies as well as the lack of toxicity, combined with the ability of the therapeutic to concentrate in areas with increased inflammation and spare healthy tissues, amixicile is a highly desirable treatment method that has the potential to change the way periodontal diseases will be treated.

## Materials and Methods

### Bacterial strains and culture conditions

Microbial strains *Porphyromonas gingivalis* W83^53–55^, *Porphyromonas gingivalis* 33277 (ATCC), *Prevotella intermedia* 17^34, 56^ (Leung Lab), *Prevotella intermedia* OMA 14, *Aggregatibacter actinomycetemcomitans* 33384 (ATCC), *Fusobacterium nucleatum* 25586 (ATCC), *Tannerella forsythia* 43037 (ATCC), and *Streptococcus gordonii* 10558 (ATCC) were maintained in an artificial atmosphere (composed of 80% N, 10% H, and 10% CO_2_) at 37 °C using a Coy anaerobic chamber (Ann Arbor, MI). Bacterial strains were cultured on blood agar plates (BAP) and in brain heart infusion (BHI) broth containing 5 µg/mL hemin (Sigma) and 0.2 µg/mL menadione (vitamin K). Additionally, 5% Fetal Bovine Serum (ΔFBS) and 10 µg/mL N-acetyl muramic acid (Sigma) was added to *T*. *forsythia* cultures. All media were pre-equilibrated anaerobically for at least 12 hr prior to being used in our experiments.

### Mono- and multispecies growth studies

Bacteria from blood agar plates were used to establish broth cultures. The bacteria were grown for 48 hr and then used as inoculum for an overnight (O/N) culture. That culture was then used to inoculate aliquots of BHI broth supplemented with various concentrations of amixicile (initial culture OD_600_ of 0.1 and containing amixicile at 0, 1, 5 or 10 µg/mL). Clindamycin, an antibiotic known to be an effective inhibitor for all bacteria used in our study, was used as a positive control for inhibition of bacterial growth. Bacterial growth was monitored by measuring the absorbance at OD_600_ for up to 96 hr. For the multispecies culture experiments, all bacterial species were added at the same time, at an OD_600_ of 0.05 and were incubated with various concentrations of amixicile or metronidazole for 48 hr. To test the effect of saliva and serum on the ability of the antimicrobial to inhibit bacterial growth, BHI supplemented with 10% filtered human saliva (Lee Biosystems) or 10% filtered human serum (Valley Biomedical) were used to prepare both mono- and multispecies cultures.

### DNA isolation and qPCR

An OD_600_ of 1 was used for DNA isolation from the above cultures. Cell pellets were resuspended in 50 mM EDTA containing 10 mg/mL lysozyme and 100 U/mL mutanolysin (Sigma) and incubated at 37°C for 1 hr. The DNA pellets were rehydrated at 65°C for 1 hr in rehydration buffer. DNA was isolated using the Wizard Genomic DNA purification kit (Promega) according to manufacturer’s instructions. The DNA was then used to quantify the presence of bacterial species in the various samples using a 7500 Fast Real-time PCR machine (Thermo-Fisher). Purified DNA (1 µL) and species-specific primers were added to Fast SYBR Green Mastermix (Thermo-Fisher) and run using standard cycle conditions: 95°C for 20 sec (1 cycle); 95°C for 3 sec, 60°C for 30 sec (40 cycles). The species-specific 16S gDNA primer sequences used in this study are shown in Supplemental Table 1. The cycle threshold (Ct) data were collected and then converted to absolute fold change.

### Saliva coating of plates for biofilm experiments

Human saliva was obtained from Lee Biosolutions. Saliva (10 mL) was diluted 1:2 in PBS and centrifuged at 5,500 rpm for 15 min at 4 °C. The supernatant was collected and a proteinase inhibitor cocktail tablet (Roche #11836170001) was added, then the solution was filter-sterilized through a 0.22 µM filter (Millex). The conditioned saliva was used to coat wells of 24-well tissue culture-treated plates (Falcon) at 37°C for 30 min and residual saliva was washed off the wells using sterile PBS.

### Crystal violet biofilm assays

The formation of monospecies biofilms was determined using crystal violet staining. O/N bacterial cultures were diluted with fresh BHI to an OD_600_ of 0.1 and then 1 mL was transferred to each well of the 24-well plate. These plates were incubated anaerobically for 24 hr. Media was replaced with BHI containing 0, 5 or 10 µg/mL amixicile for an additional 24 hr. Wells were washed with PBS and stained with crystal violet. Wells were then washed with PBS and dried. 30% acetic acid was then added to each well and the absorbance was read at OD_550_ using a BioTek Synergy HI Hybrid Plate Reader. *T*. *forsythia* was allowed to grow for 96 hr due to its slow growth rate, with amixicile added for the last 24 hr.

### Multispecies biofilms

Conditioned saliva was used to coat 12-well tissue culture-treated plates. Wells were washed with PBS to remove residual saliva and BHI broth was added to the wells. The wells were inoculated with 0.05 OD_600_ of *S*. *gordonii* from an O/N culture. After 24 hr, wells were washed with PBS and fresh media was added. O/N cultures of *P*. *gingivalis*, *P*. *intermedia*, *A*. *actinomycetemcomitans*, *F*. *nucleatum*, and *T*. *forsythia* were added to the wells at an OD_600_ of 0.05. Fresh media was added every 24 hr for 3 additional days. On the fourth day, BHI containing no drug, and BHI supplemented with 10 µg/mL, 20 µg/mL, and 50 µg/mL amixicile or metronidazole was added to the wells and replaced with fresh antibiotic-containing media every 24 hr up to nine days. Wells were washed with PBS and the remaining biofilm was harvested for DNA isolation and qPCR analysis.

### LIVE/DEAD staining

Overnight cultures were diluted to an OD_600_ of 0.5 in 3 mL of BHI broth containing 0, 1, 5, or 10 μg/mL amixicile. Clindamycin and metronidazole were used as positive controls at 10 μg/mL. At time points 0, 2, and 24 hr, 50 μL of each sample was transferred to a 96-well plate and mixed with 50 μL Live/Dead BacLight Bacterial Viability Kit components (Invitrogen). Further steps were done following manufacturer’s protocol. Briefly, plates were incubated aerobically in the dark for 15 min. Cells were visualized by fluorescence microscopy on the Image Express Micro XLS (Molecular Devices). Images were taken at 60X. All live and dead cells present in the view (numbers are given in Fig. 9) were manually counted to determine total cells and percent live/dead. A representative portion of each original image is shown in Fig. 9.

### HOKs and HUVECs colonization assays

Primary Human Oral Keratinocytes (HOKs, ScienCell) and Primary Human Umbilical Vein Endothelial Cells (HUVECs, Lifeline Cell Technology) were maintained at 37°C with 5% CO_2_ using oral keratinocyte medium (ScienCell) and basal media (Lifeline), respectively. For colonization experiments, HOKs and HUVECs were placed under anaerobic conditions at 37°C with pre-equilibrated media. Oral bacteria were added to monolayers of HOKs or HUVECs for 30 min anaerobically at an MOI of 100:1 in a 12-well tissue culture plate. Wells were washed three times with sterile PBS and media was then replaced with media containing no drug, 10 µg/mL amixicile, or 10 µg/mL metronidazole for 24 hr. Cells from the 12-well plate were lysed to release internalized bacteria and the mixture was spread on blood agar plates to determine colony forming unit (CFU) counts.

### Microscopy analysis

Briefly, oral bacteria was grown to mid-log phase and stained with BCECF-AM 0.2 mM solution to a final concentration of 2 µM. The culture was then incubated at 37°C in the dark for 30 min. Cells were pelleted and washed with PBS to remove residual BCECF-AM dye. HUVECs monolayers grown on 8-chamber glass slides were infected at an MOI of 100:1 and then incubated at 37°C for 30 min to allow bacterial binding and internalization. Non-attached bacteria were then washed off with PBS. Amixicile- or metronidazole-supplemented media (antibiotic concentration at 10 µg/mL) was then added and cells were incubated for 20 hr. The slides containing infected HUVECs were then washed 3x with PBS. They were incubated in 4% paraformaldehyde for 10 min, and washed 3x with PBS. 0.2% Triton X-100 was added for 10 min and the slides were again washed 3x with PBS. The cells were then stained with TRITC Phalloidin for 45 min followed by a final wash with PBS. Finally, Vectashield mounting medium with DAPI was added and the cover slides were sealed using nail polish. Images were taken via LSM 710 confocal microscope (Zeiss).

### Bioinformatics analysis

The *P*. *gingivalis* PFOR 3D homology model was constructed using the one-to-one threading option of the Phyre2 web portal for protein modeling, prediction, and analysis^57^. The homology model utilized the crystal structure of the *D*. *africanus* crystal structure (PDB id: 2C3O) as a template. All models and crystal structures were visualized using UCSF chimera^58^. Clustal-omega (part of the EMBL-EBI online resources) was utilized to align multiple PFOR protein sequences and generate the sequence identities between the selected bacteria^59^.

### Statistical Analysis

Graphpad Prism 5.0 was used to analyze the results with ANOVA. All experiments were performed in triplicate unless otherwise noted. P values for statistical significance were calculated as follows: *p < 0.0332, **p < 0.0021, ***p < 0.0002 and ****p < 0.0001 compared to the untreated control^26, 29, 51^.

## Electronic supplementary material

Supplementary information
