# Supplementary material for: Amixicile, a novel strategy for targeting oral anaerobic pathogens
Source: Sci Rep. 2017 Sep 5;7:10474. doi: 10.1038/s41598-017-09616-0 (PMC5585216; doi:10.1038/s41598-017-09616-0)
Supplement: Supplementary file 1 — Supplementary information [file 41598_2017_9616_MOESM1_ESM.pdf]

Amixicile, a novel strategy for targeting oral anaerobic pathogens

Authors

Justin A. Hutcherson[1+], Kathryn M. Sinclair[1,2+], Benjamin R. Belvin[1,3], Qin Gui[1], Paul S. Hoffman[4] and Janina P. Lewis[1,2,3*]

Philips Institute of Oral Health Research[1], Department of Microbiology and Immunology[2], Department of Biochemistry[3] Virginia Commonwealth University, Richmond, Virginia, USA; Department of Medicine, Division of Infectious Diseases and International Health, University of Virginia[4], Charlottesville, Virginia, USA

[+]these authors contributed equally to this work

Supplemental information

Supp. Table 1 qPCR primer list. Ordered from Integrated DNA Technologies.

Supplemental Material:

Bacterial species	Orientation	Sequence (5'-3')
Porphyromonas gingivalis W83	F	ACGGGAATAACGGGCGATAC
	R	CTCAGGTTTCACCGCTGACT
Porphyromonas gingivalis ATCC 33277	F	GGTGCGTAGGTTGTTCGGTA
	R	TCCTGTTTGATACCCACGCC
Prevotella intermedia 17	F	CCATCAGGTTATGCTGGGCA
	R	GTTGCAGACCTCAGTCCGAA
Aggregatibacter actinomycetemcomitans ATCC 33384	F	GTGGGGAGCAAACAGGATTAG
	R	CCTAAGGCACAAACCCATCTC
Fusobacterium nucleatum subs. nucleatum ATCC 25586	F	TCGTGTCGTGAGATGTTGGG
	R	TCACGGCTTTGCAACTCTCT
Tannerella forsythia ATCC 43037	F	AGGATGACTGCCCTATGGGT
	R	AAGCGACAAACTTTCACCGC
Streptococcus gordonii ATCC 10558	F	GCAATTGCACCACTACCAGA
	R	TGCTCGGTCAGACTTTCGTC

Supplemental Figure 1

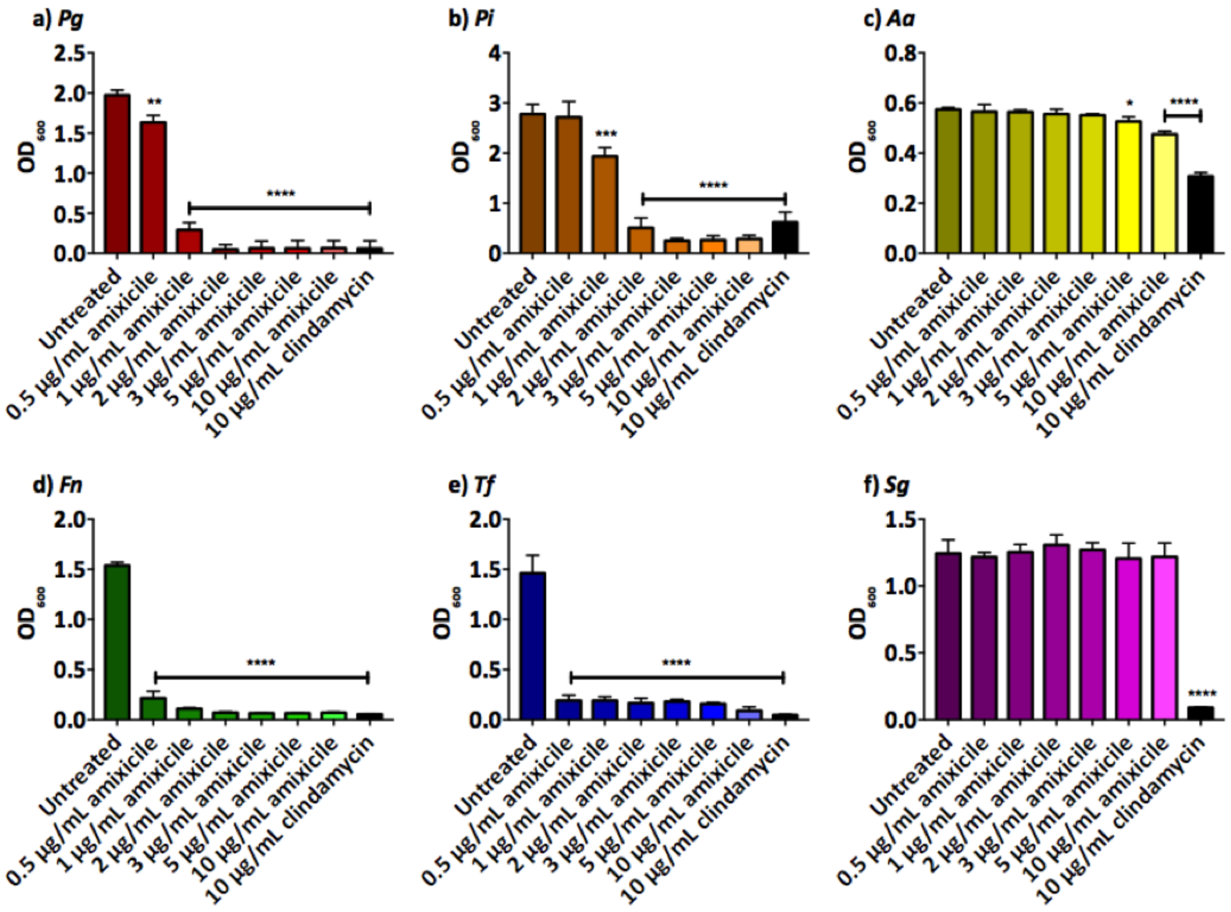

Supplemental Figure 1. Dose dependent inhibition of anaerobic bacteria by Amixicile. Bacterial strains: (A) *P. gingivalis* W83, (B) *P. intermedia* 17, (C) *A. actinomycetemcomitans* 33384, (D) *F. nucleatum* 25586, (E) *T. forsythia* 43037, and (F) *S. gordonii* 10558 were cultured in BHI broth with or without varying concentrations of amixicile from 0 to 10 µg/mL and the OD_{600} was measured at 24 hr. *T. forsythia* was measured at 96 hr due to its slow growth. Clindamycin (10 µg/mL) was used as a positive control. Results are expressed as mean ± SD. **p*<0.0332, ***p*<0.0021, ****p*<0.0002 and *****p*<0.0001 compared to the untreated control.

Supplemental Figure 2

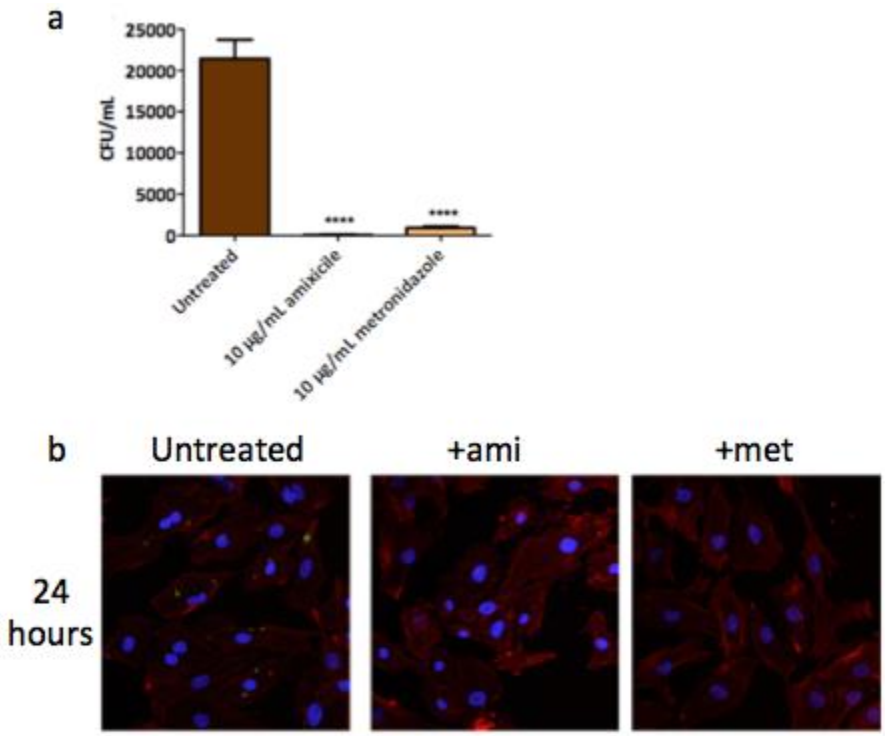

Supplemental Figure 2. Amixicile inhibits bacterial colonization of HUVECs. HUVECs were challenged with oral bacteria at an MOI of 1:100 for 24 hr with and without the addition of amixicile or metronidazole. (a) CFU counts of colonization at 24 hr as observed on blood agar plates seven days post-infection. ***p<0.001 compared to untreated control group. (b) Confocal images representing HUVECs infected with *P. intermedia* 17 for 30 min and then treated with amixicile (+ Ami) or metronidazole (+ Met) for 24 hr. Bacteria are stained in green. The cytoskeleton of HUVECs is shown in red and the nuclei in blue.
